# Contralateral dissociation between neural activity and cerebral blood volume during recurrent acute focal neocortical seizures

**DOI:** 10.1111/epi.12726

**Published:** 2014-07-22

**Authors:** Sam Harris, Luke Boorman, Michael Bruyns-Haylett, Aneurin Kennerley, Hongtao Ma, Mingrui Zhao, Paul G Overton, Theodore H Schwartz, Jason Berwick

**Affiliations:** * Department of Psychology, University of SheffieldSheffield, United Kingdom; † Department of Neurological Surgery, Brain and Mind Research Institute, Brain and Spine Center, Weill Cornell Medical College, New York Presbyterian HospitalNew York, New York, U.S.A

**Keywords:** Neurovascular coupling, 4-Aminopyridine, Electrophysiology, Optical imaging spectroscopy

## Abstract

**Objective:**

Whether epileptic events disrupt normal neurovascular coupling mechanisms locally or remotely is unclear. We sought to investigate neurovascular coupling in an acute model of focal neocortical epilepsy, both within the seizure onset zone and in contralateral homotopic cortex.

**Methods:**

Neurovascular coupling in both ipsilateral and contralateral vibrissal cortices of the urethane-anesthetized rat were examined during recurrent 4-aminopyridine (4-AP, 15 mm, 1 μl) induced focal seizures. Local field potential (LFP) and multiunit spiking activity (MUA) were recorded via two bilaterally implanted 16-channel microelectrodes. Concurrent two-dimensional optical imaging spectroscopy was used to produce spatiotemporal maps of cerebral blood volume (CBV).

**Results:**

Recurrent acute seizures in right vibrissal cortex (RVC) produced robust ipsilateral increases in LFP and MUA activity, most prominently in layer 5, that were nonlinearly correlated to local increases in CBV. In contrast, contralateral left vibrissal cortex (LVC) exhibited relatively smaller nonlaminar specific increases in neural activity coupled with a decrease in CBV, suggestive of dissociation between neural and hemodynamic responses.

**Significance:**

These findings provide insights into the impact of epileptic events on the neurovascular unit, and have important implications both for the interpretation of perfusion-based imaging signals in the disorder and understanding the widespread effects of epilepsy.

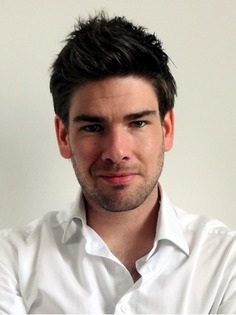

Impaired neurovascular coupling has recently been described in pathologic states such as epilepsy, which may result in altered cortical function and render interpretation of diagnostic neuroimaging signals challenging.^1^–^3^ Similarly, the epileptic event itself can also alter cortical function. Status epilepticus (SE) results in cortical damage locally, in the area of the epileptic discharges, as well in more remote brain regions.^4^ Likewise, chronic epileptic events may result in widespread cognitive and behavioral disorders.^5^ It is notable that although such effects have been ascribed to excitotoxic injury, cerebrovascular dysfunction has also been considered a potential, albeit somewhat controversial, parallel etiology,^6^ and the role of neurovascular uncoupling in widespread cortical dysfunction in epilepsy is unclear.

Currently, blood oxygenation level–dependent (BOLD) functional magnetic resonance imaging (fMRI) holds great promise as a noninvasive clinical tool for studying brain function, such as during the localization of epileptogenic tissue and adjacent eloquent cortex in medically intractable epilepsy. However, the implicit assumption, under normal conditions, of linearity in neurovascular coupling (i.e., increases in neural activation lead to proportional increases in cerebral perfusion) when interpreting these signals,^7^ may not apply to epileptic states where evidence for this is equivocal, both in the seizure focus and in remote brain regions.^2^,^3^,^8^–^11^ In turn, although there is evidence in cortex that decreases in cerebral perfusion (and the BOLD signal) may reflect neural deactivation under normal conditions,^12^,^13^ they have also been paradoxically associated with increases in neural activity in the caudate–putamen in an animal model of human absence epilepsy.^14^ This underscores the need for careful interpretation of perfusion-based imaging signals in epilepsy and further study of neurovascular coupling in the disorder.

We have recently developed the 4-aminopyridine acute model of focal neocortical seizures used frequently in our laboratory^2^,^3^ to closely mimic SE, in which ictal discharges evolve spontaneously and persistently (>45 min), and recur with small (<10 s) interseizure intervals.^1^ Here, we employ this model to examine widespread neurovascular coupling during recurrent seizures in vibrissal cortex and contralateral hemisphere of the urethane-anesthetized rat.

## Materials and Methods

### Animal preparation and surgery

All procedures were approved by the UK Home Office under the Animals (Scientific procedures) Act of 1986. Female hooded Lister rats (n* *=* *5, 250–350 g; Charles River, Margate, Kent, United Kingdom) were kept in a 12-hour dark/light cycle environment at 22°C, with food and water provided ad libitum. Animals were anesthetized with urethane (1.25 g/kg, i.p.) and atropine administered subcutaneously (0.4 mg/kg) to reduce mucous secretions during surgery. A homoeothermic blanket (Harvard Apparatus, Edenbridge, United Kingdom) and rectal probe were used to maintain core body temperature at 37°C. Animals were tracheotomized to allow artificial ventilation with pressurized room air and monitoring of end-tidal CO_2_. Blood-gas and end-tidal CO_2_ measurements were used to adjust ventilator parameters and maintain the animal within normal physiologic limits. Cannulations of the left femoral artery and vein were performed to allow arterial blood pressure measurement and maintain normotension (100–110 mm Hg) through infusion of phenylephrine (0.13–0.26 mg/h). The skull overlying stereotaxic coordinates 2 mm anterior to lambda to 2 mm anterior of bregma, and from 1 to 6 mm from midline, were thinned to translucency bilaterally so as to allow visualization of somatosensory cortices and surface vasculature, and to enable optical imaging. This is a technique used commonly in our laboratory, which preserves a largely intact central nervous system (with the exception of the small perforation made by the microelectrode) and minimizes physiologic noise (i.e., cerebral movement artefact), while providing high-resolution optical imaging data by minimizing diffuse light scattering by the skull.^1^,^13^,^15^,^16^ A thin layer of cyanoacrylate glue was then applied over these regions to reduce optical specularities from the brain surface during imaging. Animals were sacrificed following experimentation through intravenous overdose of pentobarbital.

### Epilepsy model and protocol

4-Aminopyridine (4-AP; Sigma-Aldrich, Gillingham, United Kingdom,15 mm, 1 μl) was used to elicit focal seizure-like discharges^1^–^3^ in the right vibrissal cortex (RVC). 4-AP blocks A-type, D-type, and delayed rectifier potassium channels that facilitate synaptic transmission at both excitatory and inhibitory synapses by slowing of action potential repolarization. Following a 60 s baseline recording period, we infused 4-AP at a depth of 1,500 μm via a fluidic port on the multichannel microelectrode over a 5 min period (0.2 μl/min) using a 10 μl Hamilton syringe and syringe pump (World Precision Instruments Inc., Sarasota, FL, U.S.A.). Recordings were made for 45 min following 4-AP infusion onset. No changes in mean arterial blood pressure or increased resistance to ventilation were observed following 4-AP infusion.

### Two-dimensional optical imaging spectroscopy and bilateral localization of somatosensory cortices

Two-dimensional optical imaging spectroscopy (2D-OIS) was used to produce 2D images of cerebral blood volume (CBV) over time. This technique has been described in detail previously,^15^ and as such will be described only briefly here. The brain was illuminated at four wavelengths (495 ± 31, 559 ± 16, 575 ± 14, and 587 ± 9 nm, full-width half-maximum) using a Lambda DG-4 high speed filter changer (Sutter Instrument Company, Novata, CA, U.S.A.), with image data recorded using a Dalsa 1M30P camera (Dalsa, Billerica, MA, U.S.A.) and subjected to spectral analysis.^15^ This technique produces spatiotemporal measures of oxygenated hemoglobin (HbO) and deoxygenated hemoglobin (Hbr) concentration, the product of which provides a measure of total hemoglobin (Hbt) concentration, which can be further interpreted as CBV, under the reasonable assumption of a constant hematocrit.^17^ To promote brevity, we focus on, and refer to, CBV as our hemodynamic measure throughout the following text. Bilateral somatosensory vibrissal cortices in each subject were identified by localizing evoked hemodynamic responses to electrical stimulation of each mystacial pad, as previously described in detail.^1^,^15^ A 16-channel infusion electrode containing 4-AP was then implanted perpendicular to the RVC at a depth of 1,500 μm under micromanipulator and microscope control. A second 16-channel electrode (without infusion port) was inserted identically into left vibrissal cortex (LVC). Multichannel electrodes (16 channels with 100 μm spacing; Neuronexus Technologies, Ann Arbor, MI, U.S.A.) were coupled to a preamplifier and data acquisition device (Medusa BioAmp and RZ5; TDT, Alachua, FL, U.S.A.).

### Neural recordings and analysis

Bilateral 16-channel neural data were sampled at 24 kHz. Raw electrophysiology data were band pass filtered between 0.1 and 300 Hz and down-sampled to 1.2 kHz to yield local field potential (LFP) data. Multi-unit activity (MUA) measures were obtained by band-pass filtering raw electrophysiology data, using a 500th order finite impulse response (FIR) filter between 300 and 3,000 Hz and full-wave rectification. Spikes were subsequently detected using a previously described thresholding technique,^18^ with any consecutive spike separated by <1 msec disregarded so as to account for possible refractory periods. A sliding temporal window of 10 msec moving in 1 msec steps was then used to determine spike rate (MUA). In each subject, LFP and MUA data from each channel were binned into 30 s windows beginning 60 s prior to 4-AP infusion, and summed absolute activity calculated in each bin. Binned data were subsequently normalized to the mean absolute activity during the two preinfusion bins (i.e., 60 s baseline period). Data from channels corresponding to cortical layers 2/3, 4, 5, and 6, were subsequently averaged according to previously published anatomic data by our laboratory.^19^

### Hemodynamic data

We quantified continuous hemodynamic responses in bilateral vibrissal cortices during recurrent seizures by selecting two circular regions of interest (ROIs) centered on each electrode with radius 2,250 μm. We disregarded a circular area of radius 250 μm nearest the center to avoid noise artifacts due to the electrode. Time series of all pixels in each ROI were averaged and, as with neural metrics, summated in 30 s bins beginning 60 s prior to 4-AP infusion. Binned data were subsequently normalized to the mean total hemoglobin concentration (i.e., CBV) during the preinfusion baseline and set at 104 μm.^17^

## Results

**Figure 1:**
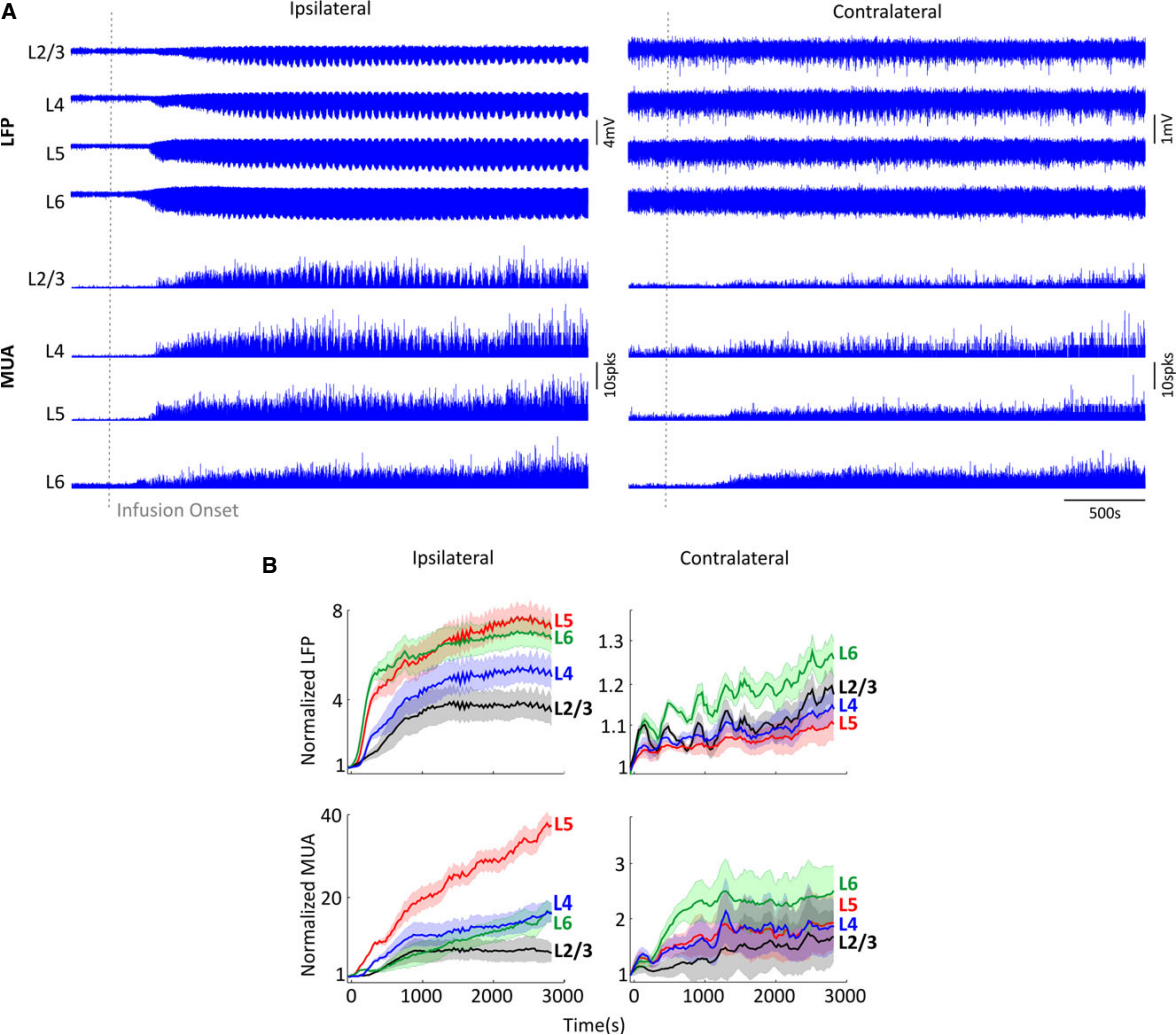
Bilateral neural responses during 4-AP–induced recurrent seizures. (A) Bilateral laminar LFPs and MUA in vibrissal cortices ipsilateral and contralateral to SOZ, in a representative animal. Gray vertical dashed line denotes 4-AP infusion onset. (B) Normalized bin-averaged (n = 5) bilateral LFPs (top) and MUA (bottom) in vibrissal cortices ipsilateral (left panels) and contralateral (right panels) to SOZ across laminae ( layer 2/3 in black, layer 4 in blue, layer 5 in red, and layer 6 in green). Shaded error bars are standard error of the mean (SEM).

4-AP infusion into RVC produced recurrent local seizure-like discharges as described previously.^1^ Increases in both LFPs and MUA were first observed shortly after infusion in layer 6 (presumed depth of induced epileptic focus) and subsequently in overlying laminae, indicating propagation of epileptiform activity (representative example shown in Fig.1A, left panel). Analysis of averaged data (Fig.1B, left panels) revealed LFP and MUA increases to be most pronounced in layer 5, as determined by the largest mean gradient (LFP, 0.06 ± 0.01; MUA, 0.38 ± 0.13; n* *=* *5). In contrast, averaged LFPs and MUA contralateral to the 4-AP infusion site exhibited relatively smaller increases in activity, which were most evident in layer 6 but not significantly laminar-specific (LFP, 0.003 ± 7 × 10^−4^; MUA, 0.02 ± 0.01; n* *=* *5) (Fig.1B, right panels).

**Figure 2:**
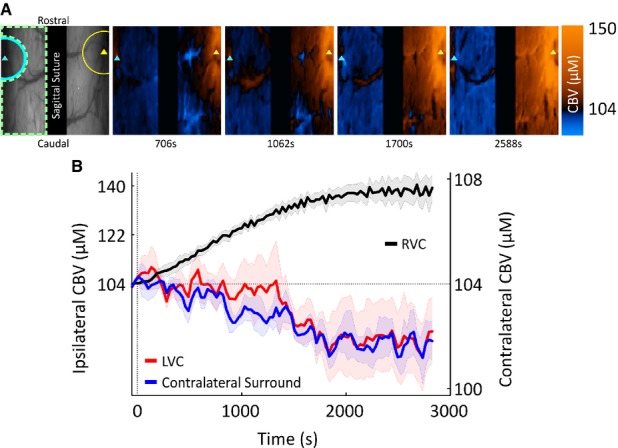
Bilateral CBV responses during 4-AP–induced recurrent seizures. (A) Far left, digital image of cortical surface showing site of implantation of 4-AP infusion electrode (yellow triangle) in RVC and additional electrode in contralateral LVC (cyan triangle) in the same subject as Figure1A. Representative ROIs are shown demarcating RVC (yellow semicircle), LVC (cyan semicircle), and contralateral surround (dashed green enclosure). Right panels, spatiotemporal changes in CBV during 4-AP–induced recurrent seizure activity (increases and decreases denoted as red and blue color maps, respectively) in the same subject. (B) Bin-averaged (n = 5) CBV time courses taken from RVC (black), LVC (red), and contralateral surround (blue) ROIs. Shaded error bars are SEM.

Infusion of 4-AP also generated a robust increase in CBV near the infusion electrode that was encompassed by a surround “negative” region associated with a CBV decrease, as described previously.^2^,^3^,^20^ CBV increases in RVC became progressively more spatially diffuse, thus overwhelming negative surround CBV regions and eventually invading the entirety of the visible right hemisphere (Fig.2A, in a representative subject). On average, CBV in RVC gradually increased following 4-AP infusion and subsequently plateaued, with an approximately 34% (104 ± 0.1 to 139.3 ± 4.7 μm) change from baseline at termination of recording (Fig.2B, n = 5).

**Figure 3:**
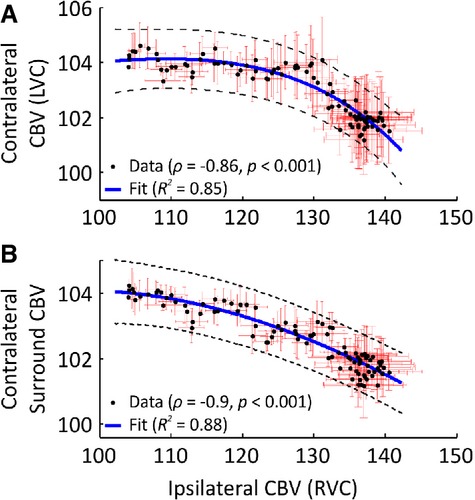
Tight relationship between bilateral CBV responses during 4-AP–induced recurrent seizures. (A) Comparison of averaged CBV responses (n = 5), ipsilateral (RVC), and contralateral (LVC) to SOZ showing a nonlinear negative correlation (Spearman's ρ = −0.86, p < 0.001). Exponential regression line (blue) fitted using nonlinear least squares (R^2^ = 0.85). (B) Nonlinear negative correlation between averaged CBV responses (n = 5) in RVC and contralateral surround region (Spearman's ρ = −0.9, p < 0.001). Best-fit exponential regression line (blue) applied as above (R^2^ = 0.88). Dashed black lines denote 99% confidence bounds. Red error bars are SEM.

Of interest, decreases in CBV were observed over large areas of the visible contralateral hemisphere with other discrete regions, primarily in the vicinity of LVC and nearby draining veins, associated with CBV increases (Fig.2A, in a representative subject). These contralateral CBV decreases intensified over time and occupied increasingly more cortical territories, whereas increases in CBV became spatially less prevalent (also Fig.2A). Averaged data revealed a progressive decrease in CBV over time in the contralateral surround and a slower initial CBV decrease in LVC, which subsequently plateaued at a reduced level approximately 2% below baseline (Fig.2B, n = 5). Comparison of CBV increases in RVC and CBV decreases in LVC showed a significant negative correlation (Spearman's ρ = −0.86, p* *<* *0.001) between both variables, best described by an exponentially decreasing function (R^2^* *=* *0.85) (Fig.3A). Similarly, CBV increases in RVC and CBV decreases in the contralateral surround were also highly negatively correlated (Spearman's ρ = −0.9, p* *<* *0.001) and best characterized by an exponential decay function (R^2^* *=* *0.88) (Fig.3B).

**Figure 4:**
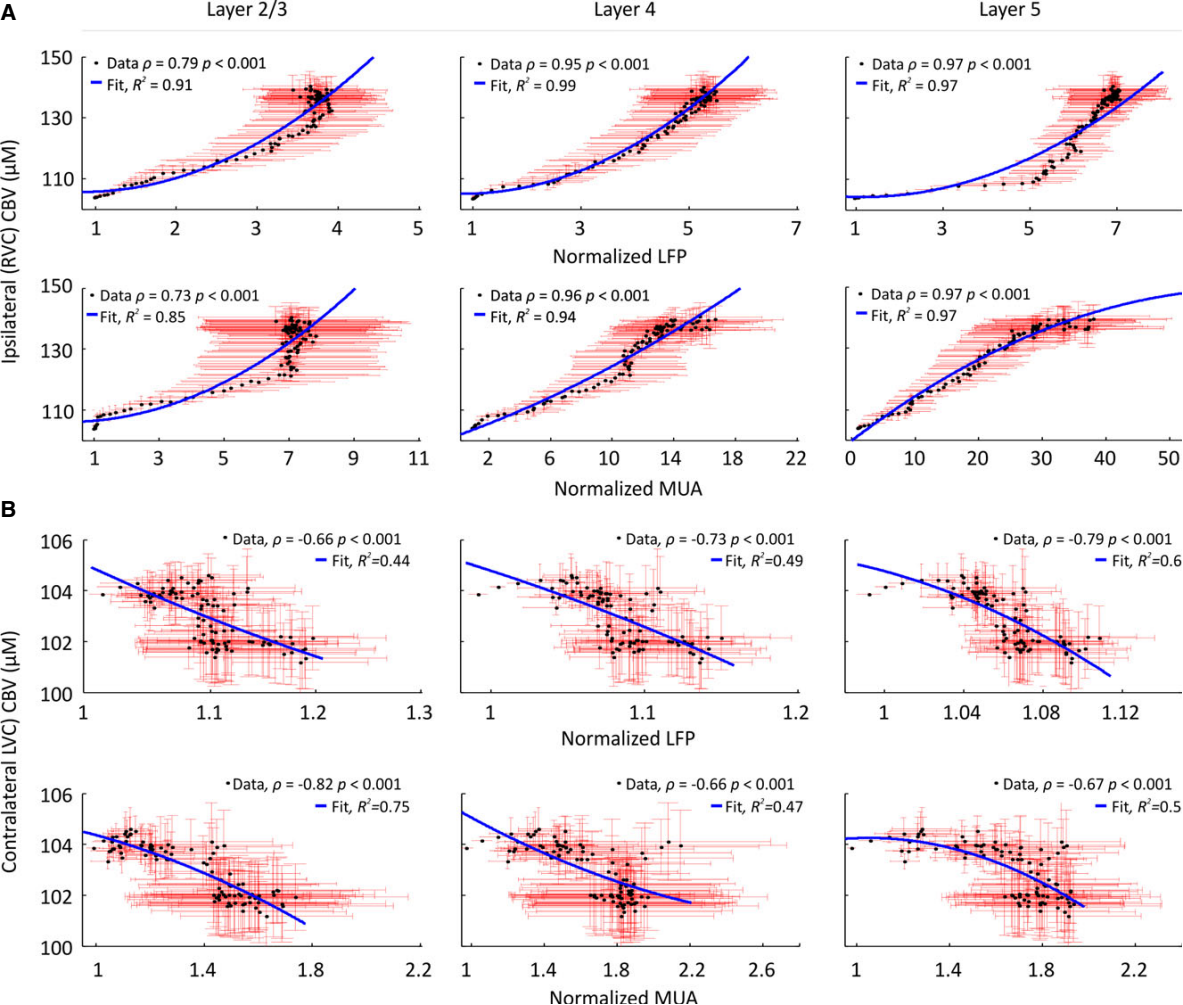
Altered neurovascular coupling in RVC and LVC during recurrent seizures in RVC. (A) Significant nonlinear correlation between averaged (n = 5) CBV responses and LFP (top) and MUA (bottom) in layers 2/3, 4, and 5 RVC. (B) Significant negative correlation between averaged (n = 5) CBV responses and LFP (top) and MUA (bottom) in layers 2/3, 4, and 5 LVC. (A–B) Second-order polynomial regression lines (blue) fitted using nonlinear least squares. Spearman correlation and fit coefficient given as inset in each case. Red error bars are SEM. Confidence intervals omitted for clarity.

Because our 2D-OIS methodology provides hemodynamic measures over cortical depths approximately <1,000 μm (i.e., layer 5),^21^ we next examined bilateral relationships between local CBV changes and LFPs and MUA in layers 2/3, 4, and 5 during recurrent seizure activity. This showed significant nonlinear correlations between CBV and neural activity across layers in RVC (most strongly in layer 5) and, of interest, significant negative correlations between CBV and neural activity across laminae in contralateral homotopic cortex (LVC) (Fig.4).

## Discussion

In summary, our findings suggest that recurrent seizures induce nonlinear neurovascular coupling ipsilateral to the seizure-onset zone (SOZ) and, intriguingly, dissociation between neural activity and cerebral blood volume in contralateral homotopic cortex. Converging evidence suggests that neurovascular coupling may be disrupted in a number of brain pathologies, such as in Alzheimer's disease,^22^ stroke,^23^ and hypertension.^24^ However, it is unclear whether neurovascular coupling is reliably altered in epilepsy.^2^,^3^,^8^–^11^ Herein, we demonstrate nonlinearity in the neurovascular coupling relationship within the SOZ during 4-AP–induced recurrent seizures, mirroring our previous discovery of nonlinear sensory-evoked neurovascular coupling during 4-AP ictal discharges.^1^ This novel finding appears to result from a CBV ceiling effect in the SOZ (approximately 140 μm), which is similar to that seen previously in our laboratory during intense vasodilation elicited by hypercapnia.^25^ It is also an important consideration when interpreting BOLD fMRI signals in epilepsy, although the observed nonlinearity may not necessarily invalidate the traditional assumption of linearity in neurovascular coupling owing to the robust monotonic relationship between neural activity and CBV. Although most BOLD fMRI paradigms in epilepsy are conducted during interictal, rather than ictal periods, interictal and ictal foci may not always be colocalized,^26^ leading to growing interest in ictal studies, as these may provide a more accurate identification of the epileptogenic zone.

Our findings in the SOZ are consistent with those of previous studies in neocortical slices showing 4-AP induced epileptiform activity to be associated with excitatory circuits in middle to deep laminae, in particular layer 5.^27^,^28^ The predisposition of layer 5 to initiate epileptiform discharges has been attributed to rich interlaminar and intralaminar connections and to a prevalence of intrinsic bursting (IB) neurons, although epileptogenesis can still be localized to this lamina in the absence of such neurons, such as in immature neocortical slices.^27^,^28^

A further novel finding of the current study is the decrease in cerebral hemodynamics, despite an increase in neural activity, in homotopic cortex contralateral to the SOZ during recurrent seizures. Although it has long been established that increases in neural activity lead to functional hyperemia and positive BOLD signals, the basis for decreases in cerebral perfusion remains under debate, with recent work demonstrating that neurovascular coupling mechanisms differ in positive and negative BOLD signal regions and to is laminar-dependent.^29^ One possible explanation for the observed vasoconstriction in our data is increased activation of astrocytes and interneurons in contralateral cortex via commissural projections originating prominently in layer 5,^30^ the locus of the greatest observed increases in neural activity in RVC. It has been shown that glutamate-induced Ca^2+^ signaling in astrocytes can lead to either vasodilation or vasoconstriction, depending on astrocytic end-foot Ca^2+^ and perivascular K^+^ levels.^31^ In addition, activation of specific subsets of inhibitory interneurons have been demonstrated to release vasoactive agents that mediate vasoconstriction in adjacent microvessels.^32^ This is consistent with previous work by our laboratory, using the same animal preparation and sensory modality as here,^13^ and others, in the anesthetized monkey visual cortex,^12^ which has shown perfusion-based signal decreases in cortex under normal conditions to be associated with robust local decreases in LFP and MUA. However, although we cannot exclude the possibility that the observed increases in LFP and MUA may be due in part to increased inhibitory neurotransmission, our finding strongly suggests that cortical hypoperfusion is not driven exclusively by neuronal suppression, at least in hemisphere contralateral to the acutely induced SOZ. Indeed, decreased cerebral perfusion can also be observed in the presence of neural activity increases in the rat caudate–putamen under normal conditions and during spike-wave seizures.^14^ Similarly, sensory-evoked decreases in cerebral blood flow despite an increase in glucose metabolism in ipsilateral somatosensory cortex has also been reported in rat.^33^ Therefore, a second, more controversial, explanation is that cerebral blood supply is redistributed from the left hemisphere to the right hemisphere to accommodate the massive CBV increase in the SOZ (i.e., vascular steal^34^). In support of this we observed inverse coupling of bilateral CBV responses during recurrent seizures, which was strongest for the contralateral surround than in LVC (Fig.3B). On a speculative note, this difference could be ascribed to a progressive redistribution of blood supply to the right hemisphere, which gradually overwhelms normal functional hyperemia in LVC induced by propagation of epileptic activity. An unknown process acting at the level of the circle of Willis may be responsible for interhemispheric redistribution of blood flow—an intriguing possibility that forms the focus of current investigation in our laboratory.

Our results confirm and extend previous reports of widespread effects on cortical function^35^ and cerebral perfusion^36^ during partial seizures. It is notable that a decrease in CBV in the hemisphere contralateral to SOZ has also been reported during secondarily generalized seizures using ictal single photon emission computed tomography (SPECT).^37^ Contralateral cerebral hypoperfusion may aid localization of the SOZ and promote impairment of cerebral function during secondarily generalized seizures.^37^ Further research is in process to elucidate whether our observation of a widespread alteration in normal neurovascular coupling could underlie reports of specific classes of interneurons showing immunopositivity for hypoxia markers in rats and patients presenting with recurrent seizures.^38^

Although our 4-AP model of epilepsy may not be a facsimile of the clinical condition, it recapitulates many of the characteristics of spontaneous chronic ictal activity in humans, which has led to its recognized utility in the study of neurovascular coupling in focal neocortical epilepsy.^1^–^3^,^16^,^20^ We do not consider the effect of 4-AP on voltage-gated potassium channels expressed on vascular smooth muscle cells to confound our observations, since the presumed corollary of this would be that of vasoconstriction (decrease in CBV) in arterioles irrigating the vibrissal cortex,^39^ which is the opposite of that presented in the SOZ. Finally, urethane anesthesia provides a persistent and stable depth of anesthesia reminiscent of sleep states and, in contrast to many general anaesthetics that are thought to enhance inhibition and/or inhibit excitation, has been demonstrated to preserve both excitatory and inhibitory synaptic transmission,^40^ leading to its common use in neurovascular coupling research.^1^,^15^,^25^ However, because our model acutely induces seizure-like discharges in the healthy ånesthetized somatosensory cortex, further research is needed to confirm our findings in the chronic epilepsy condition.

## References

[b1] Harris S, Bruyns-Haylett M, Kennerley A (2013). The effects of focal epileptic activity on regional sensory-evoked neurovascular coupling and postictal modulation of bilateral sensory processing. J Cereb Blood Flow Metab.

[b2] Ma H, Zhao M, Schwartz TH (2012). Dynamic neurovascular coupling and uncoupling during ictal onset, propagation, and termination revealed by simultaneous in vivo optical imaging of neural activity and local blood volume. Cereb Cortex.

[b3] Zhao M, Ma H, Suh M (2009). Spatiotemporal dynamics of perfusion and oximetry during ictal discharges in the rat neocortex. J Neurosci.

[b4] Wasterlain CG, Fujikawa DG, Penix L (1993). Pathophysiological mechanisms of brain damage from status epilepticus. Epilepsia.

[b5] Elger CE, Helmstaedter C, Kurthen M (2004). Chronic epilepsy and cognition. Lancet Neurol.

[b6] Fabene PF, Merigo F, Galiè M (2007). Pilocarpine-induced status epilepticus in rats involves ischemic and excitotoxic mechanisms. PLoS ONE.

[b7] Logothetis NK, Pauls J, Augath M (2001). Neurophysiological investigation of the basis of the fMRI signal. Nature.

[b8] Stefanovic B, Warnking JM, Kobayashi E (2005). Hemodynamic and metabolic responses to activation, deactivation and epileptic discharges. NeuroImage.

[b9] Hamandi K, Laufs H, Nöth U (2008). BOLD and perfusion changes during epileptic generalised spike wave activity. NeuroImage.

[b10] Mirsattari SM, Wang Z, Ives JR (2006). Linear aspects of transformation from interictal epileptic discharges to BOLD fMRI signals in an animal model of occipital epilepsy. NeuroImage.

[b11] Voges N, Blanchard S, Wendling F (2012). Modeling of the neurovascular coupling in epileptic discharges. Brain Topogr.

[b12] Shmuel A, Augath M, Oeltermann A (2006). Negative functional MRI response correlates with decreases in neuronal activity in monkey visual area V1. Nat Neurosci.

[b13] Boorman L, Kennerley AJ, Johnston D (2010). Negative blood oxygen level dependence in the rat: a model for investigating the role of suppression in neurovascular coupling. J Neurosci.

[b14] Mishra AM, Ellens DJ, Schridde U (2011). Where fMRI and electrophysiology agree to disagree: corticothalamic and striatal activity patterns in the WAG/Rij rat. J Neurosci.

[b15] Berwick J, Johnston D, Jones M (2008). Fine detail of neurovascular coupling revealed by spatiotemporal analysis of the hemodynamic response to single whisker stimulation in rat barrel cortex. J Neurophysiol.

[b16] Harris S, Ma H, Zhao M (2014). Coupling between gamma-band power and cerebral blood volume during recurrent acute neocortical seizures. NeuroImage.

[b17] Kennerley AJ, Berwick J, Martindale J (2005). Concurrent fMRI and optical measures for the investigation of the hemodynamic response function. Magn Reson Med.

[b18] Quiroga RQ, Nadasdy Z, Ben-Shaul Y (2004). Unsupervised spike detection and sorting with wavelets and superparamagnetic clustering. Neural Comput.

[b19] Devonshire I, Mayhew J, Overton P (2007). Cocaine preferentially enhances sensory processing in the upper layers of the primary sensory cortex. Neuroscience.

[b20] Schwartz TH, Bonhoeffer T (2001). In vivo optical mapping of epileptic foci and surround inhibition in ferret cerebral cortex. Nat Med.

[b21] Kennerley AJ, Berwick J, Martindale J (2009). Refinement of optical imaging spectroscopy algorithms using concurrent BOLD and CBV fMRI. NeuroImage.

[b22] Zlokovic BV (2005). Neurovascular mechanisms of Alzheimer's neurodegeneration. Trends Neurosci.

[b23] Del Zoppo G (2010). The neurovascular unit in the setting of stroke. J Intern Med.

[b24] Girouard H, Iadecola C (2006). Neurovascular coupling in the normal brain and in hypertension, stroke, and Alzheimer disease. J Appl Physiol (1985).

[b25] Kennerley AJ, Harris S, Bruyns-Haylett M (2011). Early and late stimulus-evoked cortical hemodynamic responses provide insight into the neurogenic nature of neurovascular coupling. J Cereb Blood Flow Metab.

[b26] Tyvaert L, Hawco C, Kobayashi E (2008). Different structures involved during ictal and interictal epileptic activity in malformations of cortical development: an EEG-fMRI study. Brain.

[b27] Borbély S, Halasy K, Somogyvári Z (2006). Laminar analysis of initiation and spread of epileptiform discharges in three in vitro models. Brain Res Bull.

[b28] Hoffman SN, Prince DA (1995). Epileptogenesis in immature neocortical slices induced by 4-aminopyridine. Dev Brain Res.

[b29] Goense J, Merkle H, Logothetis NK (2012). High-resolution fMRI reveals laminar differences in neurovascular coupling between positive and negative BOLD responses. Neuron.

[b30] Wise S, Jones E (1976). The organization and postnatal development of the commissural projection of the rat somatic sensory cortex. J Comp Neurol.

[b31] Girouard H, Bonev AD, Hannah RM (2010). Astrocytic endfoot Ca^2+^ and BK channels determine both arteriolar dilation and constriction. Proc Natl Acad Sci USA.

[b32] Cauli B, Tong X-K, Rancillac A (2004). Cortical GABA interneurons in neurovascular coupling: relays for subcortical vasoactive pathways. J Neurosci.

[b33] Devor A, Hillman EM, Tian P (2008). Stimulus-induced changes in blood flow and 2-deoxyglucose uptake dissociate in ipsilateral somatosensory cortex. J Neurosci.

[b34] Harel N, Lee S-P, Nagaoka T (2002). Origin of negative blood oxygenation level–dependent fMRI signals. J Cereb Blood Flow Metab.

[b35] Blumenfeld H, Rivera M, McNally K (2004). Ictal neocortical slowing in temporal lobe epilepsy. Neurology.

[b36] Van Paesschen W, Dupont P, Van Driel G (2003). SPECT perfusion changes during complex partial seizures in patients with hippocampal sclerosis. Brain.

[b37] Varghese G, Purcaro M, Motelow J (2009). Clinical use of ictal SPECT in secondarily generalized tonic–clonic seizures. Brain.

[b38] Gualtieri F, Marinelli C, Longo D (2012). Hypoxia markers are expressed in interneurons exposed to recurrent seizures. NeuroMol Med.

[b39] Horiuchi T, Dietrich HH, Tsugane S (2001). Role of potassium channels in regulation of brain arteriolar tone comparison of cerebrum versus brain stem. Stroke.

[b40] Sceniak MP, MacIver MB (2006). Cellular actions of urethane on rat visual cortical neurons in vitro. J Neurophysiol.

