# Supplementary material for: Contralateral dissociation between neural activity and cerebral blood volume during recurrent acute focal neocortical seizures
Source: Epilepsia. 2014 Jul 22;55(9):1423–30. doi: 10.1111/epi.12726 (PMC4336552; doi:10.1111/epi.12726)
Supplement: Supplementary file 1 [file epi0055-1423-sd1.pptx]

## Slide 1
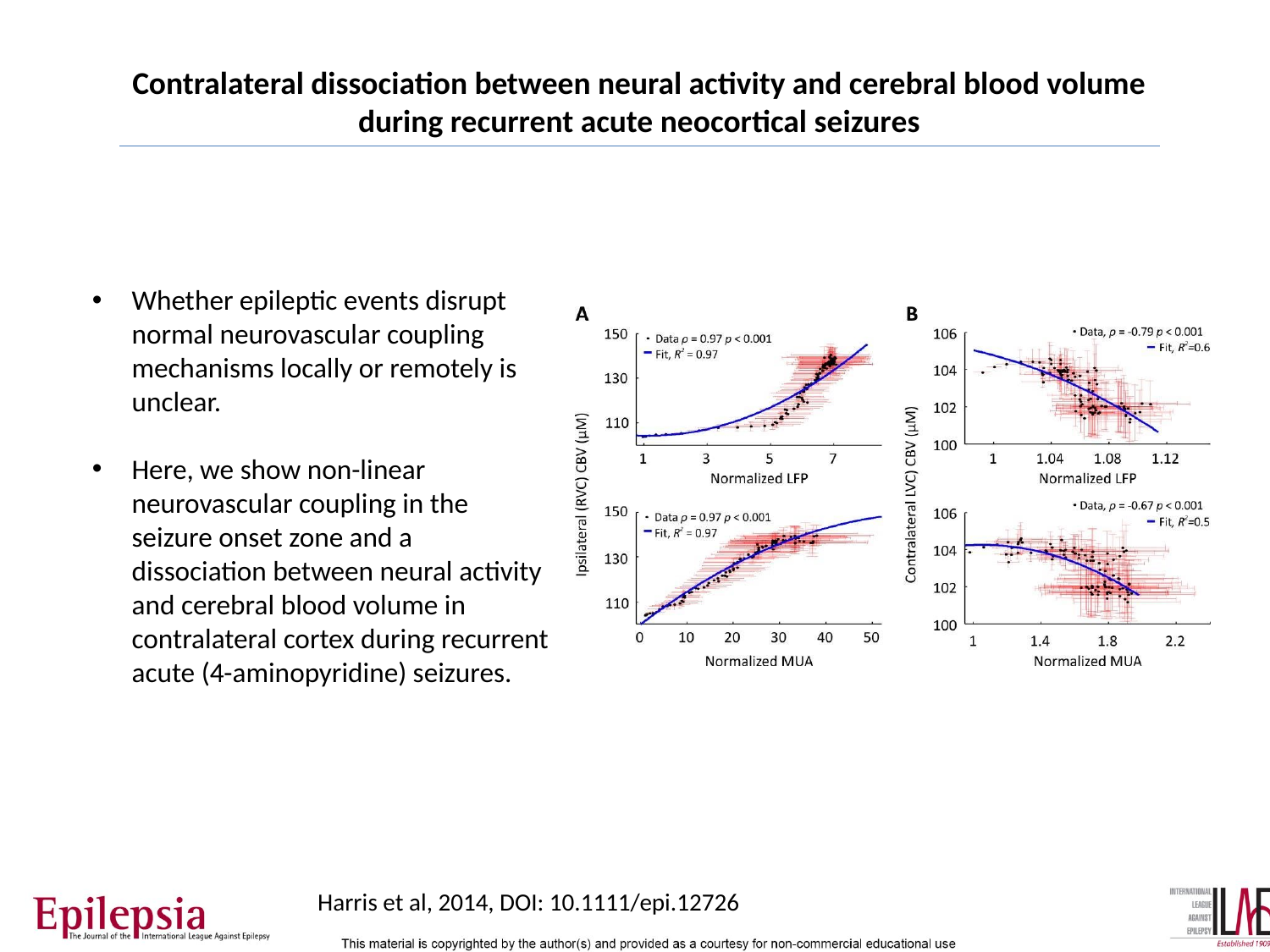

Contralateral dissociation between neural activity and cerebral blood volume during recurrent acute neocortical seizures
Whether epileptic events disrupt normal neurovascular coupling mechanisms locally or remotely is unclear.
Here, we show non-linear neurovascular coupling in the seizure onset zone and a dissociation between neural activity and cerebral blood volume in contralateral cortex during recurrent acute (4-aminopyridine) seizures.
Harris et al, 2014, DOI: 10.1111/epi.12726
